# Book Reviews

**Published:** 1815-04

**Authors:** 


### CRITICAL ANALYSIS
### *OF RECENT PUBLICATIONS*
IN THE
### DIFFERENT BRANCHES OF PHYSIC, SURGERY, &c.

*Cases of Tetanus and Rabies Contagiosa, or Canine Hydrophobia, with Remarks chiefly intended to ascertain the Characteristic Symptoms in Man and certain Brutes, and to point out the most effectual Means of Prevention.* By Caleb Hillier Parry, M. D. F. R. S. &c. &c.—8vo. pp. 218. Bath: printed for Underwood, Fleet-street.

NOTHING gives us higher satisfaction than a publication from a veteran in our art, particularly where we have reason to expect sound judgment joined to long experience. Both these are united in Dr. Parry, and no one will dispute the importance of the subjects on which he has thought proper to inform the public.

The first paper on tetanus is introduced by four cases from wounds; three proved fatal, two of which were examined after death, and, as is usual, nothing was discovered which could account for the symptoms or their fatality. Some very judicious remarks follow, but, as they are not quite satisfactory to us, we shall not scruple to offer our objections, which, if not agreeable to Dr. Parry, may afford him an opportunity of giving further information to our readers and to ourselves.

"In reality, the system on which the cause of tetanus, whatever it may be, is found to act, is evidently the muscular; not only of voluntary motion, or what Bichât terms animal life, but also of automatic or involuntary motion, called by Bichât organic life. We see the first in the facial, cervical, dorsal, abdominal, and other voluntary muscles; and the latter in the heart, the actions of which are preternaturally quick; so that, in this malady, one may fairly estimate the danger of the disease, on one hand, or the probability of the recovery, on the other, by the quickness or slowness of the pulse.

"If, however, we look for this increased action of the heart in the origin of the cardiac nerves, which, according to Le Gallois and others, is in the medulla oblongata, about the roots of the par vagum, we shall there find no change from what appears to be the natural state.

"It seems, therefore, as if the cause of this malady operated in some unknown manner on the *vis insita* of the muscles; whether we judge that power to depend on mere organization, or to be a substance, like what has been supposed to be the principle of life in other parts, acting upon a structure fitted to receive its influence; from the loss of which in the heart, that organ, finally exhausted, sinks, and the patient dies.

"But whatever may be the mode in which it is acted on, the heart, in reality, seems to be that part of the frame, which, in tetanus, first loses its vital powers.

"On an observation of this fact, we may, as I have before hinted, form a just, or at least a highly probable, conclusion as to the event of the disease. If, in an adult, the pulse by the fourth or fifth day does not reach 100 or perhaps 110 beats in a minute, I believe the patient almost always recovers. If, on the other hand, the pulse on the first day is 120 or more in a minute, few instances will, I apprehend, be found, in which he will not die. In my patient Collins, the pulse on the fifth day reached 120; but I have little doubt that the increase of quickness arose from the increase of salivation, all the other symptoms of the disease being then on the decline."

These remarks are followed by some very fair conjectures, that the successful cases met with in different collections have all been such as might have recovered under any treatment. On speaking afterwards of remedies, we are told—

"Were an instance now to occur to me, I should be disposed to try the effects of large doses, frequently repeated, of calomel and jalap, as a purgative; so as to procure very copious evacuations during several days. It was chiefly with this view that the calomel was administered in the case of Collins; and a similar practice practice seems to have been the most successful in the case of Mr. Dickinson. If, however, the pulse were at the beginning of the malady 120 or more in a minute, I would add to these remedies friction with mercurial ointment, to the amount of one or two ounces in the twenty-four hours, successively or conjointly employed, on the legs, thighs, abdomen, and arms. This latter remedy, united with drastic purgatives, might be tried, where, from the violence or situation of the disease, the patient was unable to swallow."

The author does not seem aware, that the ingenious prognosis stated by him bears very much on another too much neglected. Celsus, speaking of these complaints, says, "Ea sæpe intra quartum diem tollunt. Si hunc evaserint, *sine periculo* sunt.—*Lib.* 4, *cap.* 3. We shall not enquire what is meant by the unknown manner in which the cause of this malady operates on the *vis insita* of the muscles, nor even what is meant by the *vis insita.* That the fatality of the disease depends on the muscles of the heart partaking of the contraction is, satisfactorily we think, proved in an ingenious paper by Mr. Howship, in our 22d vol. p. 188. This, and our preceding volume, afford many very valuable cases by that able surgeon; and we regret they are unnoticed by our author. Nor can we see why Dr. Parry should omit the free use of opium in any future case, for, whether the patient who survived was cured by that remedy or not, he certainly recovered under its use. Three cases follow of tetanus in horses, which afford no particular practical instructions.

The section on Hydrophobia also commences with cases; on these, we shall be as short as possible,—the melancholy list being already very long. We shall principally attend to the dissections. In the first,

"Every part of the brain, medulla oblongata, larynx, trachea, lungs, heart, pericardium, pharynx, œsophagus, stomach, intestines, liver, gall bladder, pancreas, spleen, kidnies, and bladder, together with the meninges of the brain, and the pleura and peritonæum, was most carefully examined, both within and without; but in no part was there the least preternatural effusion, discolouration, or any deviation whatever from the healthy state.

"There having been no inflammation or pain in the wounded parts subsequently to cicatrization, they were not opened."

The following is the account of the second:

"There was no appearance of disease in the fauces, or in the upper part of the pharynx or trachea. At the commencement of the œsophagus, for about two inches downwards, there was, in its substance, a greater degree of lividness, than through the rest of its tract; and the cellular membrane behind that portion had in it a small quantity of blood, which might possibly have been extravasated travasated before death; though not probably, as a small branch of the carotid near the part was cut through in the dissection, and did actually bleed into the adjoining cellular membrane.

"The cardiac portion of the stomach had its villous coat, and that only, of a more florid red colour than what is supposed to be usual, as if from preternatural fulness of the minute arteries; and the large venous trunks were unusually distended with blood. The redness did not extend up into the œsophagus, nor did it affect any other part of the stomach, except a small portion immediately about the pylorus. The stomach was rather larger than common, and in a lax state; and had in it about three-quarters of a pint of fluid, similar to what it usually contains. There was no appearance of disease in any other of the abdominal viscera.

"The lungs, and all the rest of the thoracic viscera, were in the natural state, except that the internal membrane of the trachea, for some inches upwards from its bifurcation, was redder than common, as if its minute arteries were injected with blood. It was not covered with any preternatural secretion.

"The medulla oblongata, and all the parts within the cranium, were of the usual appearance. The blood was every where fluid."

In the third case,

"The cellular membrane of the abdomen was so distended with air, that the epidermis seemed, when pressed on, to be in some places distant two inches or more from the muscular substance within.

"The head was unusually large. The dura mater adhered firmly to the cranium. The pia mater, and general substance of the brain, were every where somewhat more vascular and watery than is commonly observable in adults; but we doubted whether in a greater degree than is common to the age of this patient. Every part of the brain and medulla oblongata, together with the great arterial circle, was carefully examined, and was found free from disease. The ventricles were void of lymph, and the pineal gland of any thing gritty. The only observable deviation from health was in the fourth ventricle, on the anterior paries of which, at the upper part, there was a dark spotted patch as from ecchymosis, or black extravasated blood. As the ventricle was opened longitudinally from behind, this discoloured part seemed to include a space nearly as large as a silver fourpence.

"In the thorax, there was emphysema under different parts of the pleura pulmonalis, on both sides. On the right side, there was an extravasation of about two ounces of bloody fluid, but none on the left. Both lungs had in them a good deal of dark blood, but could not be said in any respect to labour under disease, except from the appearance of one small tubercle in the left lobe. The pleura costalis was every where in the usual condition. The heart was extremely hard, and strongly contracted; having in its cavities a very little clotted blood, and no disease in the valves or or coronary arteries. The tongue, pharynx, larynx, trachea, and œsophagus as low as the diaphragm, were taken out of the body; but after the closest examination, not the smallest redness could be detected in any part of them. A little thin mucous bedewed the inside of the trachea. The œsophagus was traced to the stomach, and was perfectly pale. In the stomach there was a similar appearance, except that there were two or three faint stripes of pale red about the cardiac portion, which by no means amounted to disease. The stomach contained about a spoonful of a pale pulpy liquid. The liver and all other parts were in a sound state. The bladder was full of urine."

We shall only remark, in the last case, the coincidence in the state of the heart with Mr. Howship's observation in the dissection of a tetanic case.

A case follows of imaginary hydrophobia; after which the author draws his inferences on the former three.

"All those who carefully peruse these cases, must be struck with their close coincidence as to certain symptoms; which may, therefore, be considered as the characteristics of the disease.

"In the first place, the part which is primarily affected, so as to give occasion to the symptom called hydrophobia, is not the pharynx, œsophagus, or stomach, but the upper portion of the trachea, together with other parts of the apparatus concerned in the function of respiration. The truth of this position is evident from the following circumstances:

"First. At certain periods of the disease, when it was marked with sufficient strength, two out of three of these patients swallowed solids without difficulty, or any tendency to the production of the convulsion.

"Secondly. The local convulsions were brought on, not only by swallowing, but by a quick impulse of air, such as is actually known to put out of breath, in a slighter degree, young persons, whose respiratory organs may readily be supposed to be in an irritable state.

"Thirdly. The spontaneous attacks of the convulsions, whether during sleep or waking, always began with a violent sobbing inspiration, in which the shoulders were generally preternaturally elevated, as in asthma, and the abdomen strongly protruded by the forcible depression of the diaphragm.

"Fourthly. This state was still more strongly marked, when the convulsion was brought on by the attempt to swallow liquids, by the mere contact of them with the fauces, before any attempt to swallow them, by the apprehension of them, or by the strong impulse of air: in either of which cases the power of respiration seemed suspended, or very irregularly performed, and the patient experienced a sense of strangulation, which shewed itself by an apparent constriction of the rima glottidis, and in the corresponding gestures of the patient."

"Another circumstance, characteristic, as it should seem to me, me, of this disease, is an inordinate action of the voluntary muscles; whether from a perverted function of the brain, or a want of power in the muscles themselves precisely to obey the will, it may be hard to determine. The muscles seem always to over-act the intention of the mind."

"A third characteristic circumstance is the peculiar nature of the delirium; different, certainly, from that which occurs in any other malady, with which I am conversant. The patient shall go on talking with vehemence, and for many successive hours, of past events, as if they were actually present; shall fancy objects to be different from what they really are, and shall even act on those delusions; and yet no sooner is his attention excited by questions put to him by his friends, than he shall answer them, and continue for a considerable time to converse, with a calmness of manner, and a coherency of ideas, precisely similar to those of a person in perfect health. This more or less occurred with regard to all three patients nearly to the last; and it is stated, in the case of Master English, that fourteen hours before his death, in the intervals of his convulsions, he was amusing himself with his playthings."

Some remarks follow to shew the diagnostic distinctions between the above disease and epilepsy, as well as tetanus, after which an inquiry follows concerning the origin of the poison, which is, of course, left in doubt; the author's opinion, however, is, that it is not excited *de novo*, but always by the application of saliva from a rabid animal. He seems to doubt whether the infection has been traced beyond dogs, wolves, foxes, and cats. The following is a summary of the remarks concerning the medium time from the bite to the appearance of the disease.

"I have already remarked that the poison sometimes lies long in the part without any apparent action on the constitution. If Tucker really owed his disease to the supposed bite in the month of November, 1809, seven months must have elapsed before the disease shewed itself. Galen asserts that he saw a case after a year; Avicenna speaks of six months as a possible interval; and Dr. Mead remembered one example after eleven months. The case of Stephen Bellass, by Mr. Nourse, which is a probable example of the disease, exhibits an interval of the extraordinary length of nineteen months.* Master English was taken ill fifty-two days, and Selway twenty-three days, after the bite. This last is the shortest interval which I have been able to find in any well-defined example of the disease; and exactly accords with that in the case of John Dyke, by Dr. Beddoes.† The period commonly assumed is about forty days; and a mastiff of mine, which died rabid, became ill forty days after he was seen to have been bitten

"* Philosophical Transactions, No. 445.

"† London Medical and Physical Journal, vol. xx. p. 195."

by a small terrier, which, within a quarter of an hour after, bit another dog, who also died with similar symptoms, within twenty-four hours before or after the death of my mastiff. The shortest interval, according to Mead, is fifteen or sixteen days."

The uncertainty of the duration of the disease is next judiciously dwelt upon; after which follows an important observation, too little noticed by most writers, namely, the speedy decomposition of the parts after death, and the fluidity of the blood; in other words, we should say, the rapidity with which *absolute universal death* follows the cessation of respiration. This should always be kept in view as the means of accounting for that apparent inflammation of the stomach, remarked by so many persons unaccustomed to the examination of dead bodies, but which, in reality, is only a partial solution of the villous coat of the stomach, in consequence of its becoming dead before the gastric juice contained in the secretory part is urged into the cavity. This was particularly exemplified in one of Dr. Parry's cases (Selway); though opened only eight hours after death, decomposition was advancing; and here it is remarked, there were found two or three faint stripes of pale red about the cardiac portion, which, it is afterwards observed, often occur in persons who die without any previous symptoms of inflammation in the stomach. "There cannot," proceeds our author, "be a greater mistake than to suppose, that the fever is of the inflammatory kind, &c." At present we shall only remark, that, in the case he produces to illustrate his opinion, the blood in the progress of the disease shewed an inflammatory crust, and the urine was high coloured: the latter is ascribed by Dr. Parry to the small quantity of fluid swallowed by the patient. It could not be supposed, after this bold assertion, that Dr. Parry would overlook those histories of cure by bleeding, transmitted to us from the East Indies.*

That there are difficulties attending the complete identification of the cases with rabies is certain; but we are by no means satisfied with the subsequent reasoning of our author. "It is, however, certain," says he, "that the being bitten by a healthy dog will not prevent convulsions, phrenitis, nor any of the diseases confounded with the rabies;—nay, a man may have these diseases after being bitten by a rabid dog, and not have hydrophobia."—Did any one doubt, that a man may have convulsions and phrenitis under any circumstances? But we have read well-authenticated

* See London Medical and Physical Journal, vol. xxviii. p. 381; and Ibid, vol. xxix. p. 34 & seq.

cated cases in which no symptoms of rabies have appeared in the dog, yet the bite has produced rabies, and this too in subjects who have had no suspicion of the cause of their disease. As Dr. Parry has searched a great number of records, we cannot help wondering that he should have passed over those two important ones related by Mr. Parkinson in a Journal which he has not entirely overlooked for other authorities.*

"The pulse (says Mr. P.) was frequent; the heat but little more than natural; the mouth was dry, and the tonsils slightly inflamed. She complained exceedingly of thirst, but said it was impossible to drink; for that, as I had seen, directly as the liquid touched her lips, it threw her into convulsions: I therefore promised that I would give her some drink without letting it touch her lips. This I attempted by dropping water on her tongue from a tea-spoon, she opening her lips and mouth as wide as she was able, and leaning her head back on the pillow. This succeeded for a little time, she swallowing the water, and expressing much gratification; but a drop falling aside on her lips, she became exceedingly agitated, and was with difficulty persuaded to allow me to make another attempt in a mode in which I told her I could better secure the water from dripping aside. This, however, she at length submitted to, and by dripping the water from the end of my finger on her tongue, she swallowed nearly half a glass full of water with much pleasure, and without any apparent inconvenience. I then placed some calves'-feet jelly on her tongue, in its more solid form; which, as it melted, she swallowed pleasantly, and of this she took freely.

"An opportunity offering, I enquired what had become of the little dog I had been accustomed to see at the house. She informed me, "that it had become so troublesome that it had been sent away.—" Had it," I asked, "become cross and snappish?"— "No," she said, "never—it was rather too fond, for it was perpetually leaping on her lap, and licking her hands and arms," which I observed were still rough from chaps. "Then," I said, "he never bit any of the family?"—"Never."—"Pray," I then asked, "were you ever bitten by any dog?"—Never," she eagerly answered, adding, "Why do you ask?"

"When Sir W. Blizard came, he procured her immediate removal to the London Hospital, where, to the best of my recollection, a consultation of all the medical and surgical officers was obtained, but in vain. She died, I think, within forty-eight hours from her removal. The inspection of the body, which was performed with a particular degree of minuteness and care, presented only the usual unsatisfactory appearances."

The other case is, in our opinion, not less satisfactory.

"The wounds, (we are told,) were drest with red precipitate and a terebinthinate ointment, and healed in about five days.

---

* Repository, vol. i, page 289.

But, at nearly three weeks from the infliction of the bite, I was sent for to the boy, he having had some slight feverishness the night before, which was rather increased. My son accompanied me, and we soon discovered that the dreadful malady was established. Ignorant of any measures that could be relied on, and as pyrexia, with evident inflammation of the tonsils, existed, we agreed on the experiment of taking away blood, which was done to the quantity of 6 or 7 ounces, by which a slight degree of faintness was produced, without any apparent amendment.

"The benevolent assistance of Dr. Yellowly was now requested, and immediately obtained, when, it being considered that no medicine had manifested any remedial powers in this disease, it was agreed to make trial of the effects of lead, and to endeavour to moderate the more urgent spasms by the employment of henbane. The experiment was fully made, the superacetate of lead and the extract of hyosciamus were had recourse to, but without the least advantage; the child, after suffering from every decided symptom of hydrophobia, being seized with convulsions so violent as to require two men to retain him in the bed; to which succeeded a state of quiet insensibility, lasting about half an hour, and terminating in his death, which took place on the third day from the attack.

"On the following day a most accurate inspection of the body was made with the assistance of Dr. Yellowly, Mr. G. Young, and Dr. Berger, but without discovering a single circumstance which could point out the seat, or the nature of the disease."

We are aware that Mr. Parkinson relates these cases from memory; but we have too good an opinion of his accuracy to conceive that he made up his mind too hastily, or that in so interesting a history his memory should have betrayed him in any material point. We quote the following passage that our readers may form their judgment, whether they are to accuse Dr. Parry of unreasonable scepticism, or applaud his accuracy, as the cases are all taken from our Journal.

"The case of Anne Chandler, by Dr. Powell,* appears to me to be evidently œsophagitis, and not rabies; and that of William Waters, by Dr. Pinckard,† laryngitis mixed with œsophagitis: that of William Miles, by Mr. Hicks,‡ mania from fear; and that of Elizabeth Kirk, by Mr. Borrett,§ gastritis with ulceration.

"Respecting this last case I must observe, that various disorders in the stomach, and more especially that state, in which large quantities of blood are ejected by vomiting, are, in females, often accompanied with singultus, aversion to swallowing, great inconvenience from it, and frequent paroxysms of hysteria.

---

* London Medical and Physical Journal, vol. xx. p. 201.
† Ibid. vol. xxi. p. 58. ‡ Ibid. vol. xvii. p. 272.
§ Ibid. vol. xxi. p. 268.

 "I may

"I may also advert to two other cases, by Dr. O'Donnel, supposed, I think erroneously, to be rabies. The first, that of William Honey, was a clear example of acute inflammation of the larynx and pharynx, attended with a similar state of the brain, and consequent extravasation in the ventricles. The second, that of Joseph Watson, was also one of sore throat. No dissection of this patient is, however, given in the London Medical and Physical Journal, (vol. xxix. p. 485, et seq.) from which I quote.

"In all these instances, there was dread of water, from the inconveniences attendant on swallowing it. But though the patients were bitten by dogs, there was no evidence of canine rabies; in which, as I have before observed, the spasm of the respiratory organs accompanies the contact of liquids with the inside of the fauces, before any attempt whatever is made to swallow them."

The inference from the whole is, that rabies is not an inflammatory disease, and that it is incurable; hence, Dr. Parry very properly directs us to the preventive means. Here he advises large ablutions and free excision at any period after the bite, and even at the commencement of the symptoms. His illustrations are not, however, quite satisfactory. We do not know, that the experiment of excision has ever been tried in the small-pox. The experiments of Fontana on the poison of the viper are not at all to the purpose. The poison of the viper is not a morbid poison.

"The poison, (we are told,) for ought we know, may be increased by a process equivalent to fermentation; and the part may be the reservoir, from which the constitution is successively imbibing, even until death, the *materies morbi.* We are equally ignorant of the quantity which may be necessary in order to produce death. It was found by Fontana, that the effects of the poison of the viper depended on this circumstance; so that the bite of one viper would kill a small animal, such as a fowl or a rabbit, but was insufficient to destroy a man, or even a middle-sized dog. A larger quantity killed a dog; and a still larger would, probably, have produced the same effect on a man. Perhaps, therefore, the excision of the part, even after the pains in the course of the nerves have begun, may so cut off the supply of virus, as to make that, which has been already received, inadequate to the destruction of life. I offer this merely as an hypothesis; on which, however, I would certainly act, should an opportunity, in which the operation was admissible, hereafter present itself."

Now, in morbid poisons, it should be recollected the quantity applied, if sufficient to produce any morbid effect, is of no consequence as to the future disease. The poison of the viper does not enable a poisoned animal to injure another like a morbid poison. We say nothing about fermentation, an expression we should not have expected from Dr. Parry, when speaking of the living body. Whatever may may be the cause of increased *materies morbi*, nothing of the kind exists in the poison of a viper.

Dr. Parry next proceeds to mark the symptoms of rabies contagiosa in brutes. Here he seems at issue with every one who has gone before him; we shall, therefore, only in general remark, that we are by no means convinced that, though "all animals of the same class are regulated by the same physiological laws; or, in other words, that the phænomena constituting the same functions are, in all, similar both in quality and order, differing only in their degree, conformably to the known structure of the animal, and the ends of his existence;" that, therefore, "the pathology, or phenomena of deviation from the healthy performance of the different functions, must also be regulated agreeably to the same invariable principles."

"To suppose, (says Dr. P.) that the characteristic marks of disease in brute animals are either continually varying among themselves, or essentially different from those in man, is to deny that uniformity of the laws of nature, and to assume a new set of principles, according either to our own blindness or the workings of our own distempered imaginations."

It is certain, however, that cow-pox in cows differs from the effects of the same poison in the human race, and, if Dr. Jenner is right in ascribing the origin of that poison to the grease, that it differs still more in the horse. We regret with Dr. Parry that our information is so unsatisfactory in these points; but we regret still more that we cannot follow him without, in our opinion, admitting a degree of scepticism which would confound all evidence or supersede any thing like reasoning on well-established facts. We shall leave the question concerning Mr. Gillman's pigs to that gentleman. But the history related by Mr. Surr, of the effect produced on five horses, being within the reach of our readers,* and involving the accuracy of one of our valued correspondents, seems to claim our particular attention. The following is Dr. Parry's short account of these transactions.

"In the London Medical and Physical Journal, vol. xxiii. p. 1, cases are given, by Mr. Surr, of five horses, which died nearly about the same period, after having been bitten about the nose and lips by a dog, which was, in all the cases, probably the same. Except this disposition to bite, there is no evidence that the dog was mad. Of the symptoms in one or two of these horses, a full account is given by the writer. Two of them are said to have drank freely; and neither of them experienced the smallest incon-

* London Medical and Physical Journal, vol. xxiii. p. 1.

venience venience from any application of fluids, or had any convulsions, or any other symptom whatever of rabies, such as it is seen in the human race. The only two which were examined by dissection were found to have been affected with violent inflammation of the mucous membrane of the parts leading to the lungs and stomach; which might very well have produced the symptoms, but are not usual concomitants of rabies. This inflammation was probably produced by continuity of membrane from the bitten parts; a common occurrence in erysipelatous inflammation."

Such is Dr. Parry's summary history of five horses, all bitten about the same time, and each seized with symptoms in many respects similar, about the medium of three weeks. To save our readers the trouble of comparing this with the first article in our 23d volume, we shall mark the sentences as they occur:—"There was no evidence of madness in the dog, but his disposition to bite." In most cases this is all the evidence that can be produced, and in many even this is wanted.—"Of the symptoms of one or two of these horses a full account is given by the writer." Enough is collected concerning the symptoms of each to shew a coincidence in time, and in the issue of each.—"Two of them are said to have drank freely." Have we any proof that dogs do not drink freely when their bite is venomous? but it is more to the purpose to mark, that the two who were watched with the greatest care were unable to swallow.

"The only two (continues Dr. Parry) which were examined, were found to be affected with violent inflammation in the mucous membrane of the parts leading to the stomach and lungs.—This inflammation was probably produced by continuity of membrane from the bitten part."

It is painful for us to be under the necessity of asking our readers, whether such a cause is equal to such an effect? The horse, of which we have the first history, had the bitten part cut out; the incision healed readily by the first intention, and comparatively without even a cicatrizing hardness. Being a favourite animal, and the event well known to his master, he was watched with peculiar notice. For a fortnight he continued without any sensible alteration, and even for twenty-one days without remitting his work. On the following day he was incapable of drinking, and perpetually rubbed his nose. Now, if this irritation at his nose was the effect of the previous bite, it was in perfect conformity with the laws of this morbid poison, the progress of which is to induce its diseased action at a medium of twenty-one days from the insertion. If it was not the effect of the bite, we may be at least allowed to express our surprise at the uniformity of time and other symptoms in five horses, all of

 which which Dr. Parry is willing to admit were bitten on the same day by the same dog.

In the second horse to which Dr. P. refers, the bitten part healed "without any callus." He was taken ill about the 24th day, and from that time rubbed his lip incessantly, and was unable to swallow water.

Besides the above remark, we cannot help recording an uniformity in another symptom which we are surprised Dr. Parry should not have thought worth his notice. We mean the state of the urethra, remarked by Mr. Surr in two cases, of which he gives an accurate account, during the acute symptoms and after death. The same symptom was remarked by Dr. Fothergill in Mr. Bellamy's case, which Dr. Parry has considered in his enumeration, as one of the most clear, and to which he has consequently prefixed his asterisk.

It is not without considerable unwillingness, that we have thought it our duty to remind Dr. Parry of what appears to us too great a scepticism in historical records. We felt particularly interested in this respect, on account of the number of well-authenticated cases with which our volumes abound. We have, however, confined ourselves to a single paper, as sufficient to explain our meaning. To have omitted any defence of those correspondents, who, from the best intentions, have favoured us with their communications, would ill become that gratitude which, in common with our readers, we ought to feel for the trouble they have taken, and still more for the obloquy to which they expose themselves. Nor can we help observing the danger to which all philosophical evidence is exposed, if cases so minutely related, so accurately traced, and authenticated by such respectable names as eye-witnesses, are to be disposed of with so little reserve in a publication in other respects so highly deserving attention.

We still trust, that such a publication, from such an author, will find its way to the library of every collector; for though little that is new in practice can be proposed, yet the work abounds with ingenious remarks and many important truths.

---

*A Special Report of the General Committee of the London Infirmary for Curing Diseases of the Eye; in which certain Pretensions of Sir William Adams, advanced in the Official Papers published by Order of the Hon. Directors of Greenwich Hospital, lately submitted to a Medical Committee, appointed by Government, and affecting the Rights of the Infirmary, and the Merits of the late John Cunningham Saunders, Esq. its Founder and Surgeon; are Examined and*

*and Disproved by the Correspondence of Mr. Saunders, and other Documents.* London: Published, by Order of the General Committee, by Longman and Co. 1815. pp. 45.

SUCH is the length of title to a pamphlet of less than 50 wide pages, and we heartily wish we had never seen either title or book. To render it as little irksome as possible, as well as to preserve our impartiality, we shall notice the different pieces in the order in which they occur.

The first is inscribed " At a Meeting of the General Committee of this Charity"—present, among others, the three medical officers—

" A Resolution of a Sub-Committee, dated the 21st of December, 1814, appointing the medical officers of this Infirmary a Committee to examine and to report to this General Committee, the evidence which supports the rights of this Infirmary, and the merits of the late John Cunningham Saunders, Esq. its founder and surgeon, in respect to the treatment of the Egyptian Ophthalmia, against certain claims of Sir William Adams, brought forward under the sanction of the Honourable Directors of Greenwich Hospital, and submitted to the examination of the Medical Committee,* appointed by government, having been read and confirmed; the following Report of the Medical Officers, on the matters referred to their examination, was read by the president, and deliberately considered."

Here it is impossible not to remark, that the General Committee of the Eye Infirmary is opposed to the Hon. the Directors of Greenwich Hospital; and the medical officers of the Eye Infirmary to all the first practitioners in London, whose names are thrown into a note of the report.

Next comes the Report of the Medical Committee, that is, of the medical officers of the Eye Infirmary. Whatever may be the respectability of these gentlemen, it would have appeared more decorous had they left others to make a report in which the ignorant part of the world will suspect they are interested. Are there no medical characters in the kingdom, excepting the medical officers of the Eye Infirmary, who might be competent judges from proper documents produced on this important question? Sir William too, according to the papers before us, claims the evidence of

---

" * This Committee was composed of the gentlemen whose names are subjoined:—Sir Henry Halford, Bart. M.D. F.R.S. Physician in Ordinary to the King, and to the Prince Regent; Matthew Baillie, M.D. F.R.S. Physician Extraordinary to the King; Sir Everard Home, Bart. F.R.S. Sergeant-Surgeon to the King; Henry Cline, Esq. F.R.S.; Astley Cooper, Esq. F.R.S.; John Abernethy, Esq. F.R.S."

three three medical men, the physician, (we presume,) the surgeon, and the apothecary to the Hospital, whose names are affixed. We feel every moment disposed to stop till some little interesting passage induces us to keep on, and now the credit of our Journal must be our apology.

"Amongst the formulæ kept (say these gentlemen) at that Infirmary, none was more constantly used by Mr. Saunders, at the commencement of acute ophthalmia, whether of the external or internal tunics of the eye, than a simple solution of tartar emetic, so administered as either to nauseate, or to produce full vomiting. His correct reasoning on the latter effect of this remedy will be found in his Essay on Inflammation of the Iris, which was first published in the Medical and Physical Journal, in the year 1806."

Thus, then, as the organ blower always feels his importance when the organist performs well, so we feel a little proud that our pages may claim the honour of first announcing this discovery to the world, a discovery concerning which so many oculists are disputing, and so many *reports* are current.

We copy the following to show the little ceremony paid to those names which we have hitherto always mentioned with respect; and also to show that Sir William, whatever may be his real claims, seems, by the confession or allegation of the *special* Committee of the Eye Infirmary, to have given up all title to one important discovery.

"That your Medical Committee might not shew themselves inattentive to the reference of Sir William Adams to any member of the committee appointed by government, to inquire into *his claims* to a *new* and successful mode of treating the ophthalmia in *all* its stages and varieties, one of your medical officers made a personal inquiry of Mr. Abernethy, and received his permission to state, 'that the object of the committee was to observe and report on Sir William Adams's treatment of certain patients in the *chronic or last stage* of the Egyptian ophthalmia.' Thus, then, it appears, that the object of that distinguished committee was not to inquire into a new and successful mode of treating the ophthalmia in *all* its stages, as Sir William Adams asserted; but that it was confined to that *last* stage of it, for which the official papers of Greenwich Hospital unjustly gave him the honour of discovering the cure.

"But further, in examining the words in which Sir William Adams has thought proper officially to lay his claims, we observe, that the committee are appointed by government, according to his assertion, to inquire into a *new* and successful mode of treating the ophthalmia in *all* its stages and varieties. The construction and obvious meaning of this sentence must have led us to suppose, that he persevered in declaring himself the discoverer of the method of restoring sight, by removing a certain state of the conjunctiva produced by the Egyptian ophthalmia, if the document (No. VI. in the Appendix) had not also accompanied the other statement. By this official paper, which seems to have been presented to Sir David Dundas, the late Commander-in-Chief, immediately after the death of Mr. Saunders, (being dated March 1st, 1810,) Mr. Adams assigns the discovery to Mr. Saunders."

We cannot doubt the truth of the expression imputed to Mr. Abernethy; but, without those kinds of regular documents which are signed by the parties, a report is always imperfect.

In the next page we are told that Sir William only claims the honour of having improved upon Mr. Saunders. "Your *medical officers*," continue the *special committee*, "find nothing in this alleged improvement excepting a *slight variation* in the mode of carrying into effect the principle of treatment pointed out by Mr. Saunders." Is it for medical men to consider any variation, however slight it may seem, as unimportant?

"Your committee," continue these gentlemen, "take this opportunity to remark, that *to Mr. Saunders is exclusively due* the application of the third operation, or that by solution, to the cataracts of persons born blind, even in the earliest stage of infancy; in the opinion of your committee, *one of the most valuable and splendid discoveries of modern surgery*. He first *systematically* applied the same principle to the treatment of cataract in the adult, in its different forms; but the lamented arrest of his career by death, rendered the result of his experience on this subject incomplete. His successor felt it to be no less a grateful tribute of respect to the professional character of Mr. Saunders, than a duty imposed upon him, in the situation to which he was appointed, to prosecute the opportunities, which the death of Mr. Saunders had placed in his hands, of ascertaining the applicability of the operation by solution to the cataract of the adult subject. The results of his experience confirmed those of Mr. Saunders, as stated by the editor of his work, in the Essay on Congenital Cataract, and were communicated to the profession in the 4th volume of the Medico-Chirurgical Transactions. By these, it appeared, that the operation by solution was exclusively applicable to the congenital, and soft or flocculent species of cataract."

Hard words for the gentlemen to whom they are addressed, and a somewhat complicated subject for them to understand; but extremely modest: for, by "*his successor*," it is impossible these gentlemen can mean either of themselves, unless the succession of ophthalmic knowledge can be retained, like that of the ancient prophets, by the transfer of a mantle; or, like the modern successors of St. Peter, by the choice of a conclave. The only medical succession we know of, if any can be admitted, is that of a regular instruction from a predecessor a predecessor in the same walk. Now we never heard that Mr. Saunders had any other pupils than Sir William and Mr. Stevenson. We conceive, therefore, that the latter gentleman must be meant; yet, in perusing his valuable treatise on Cataract, we find him tracing the operation alluded to much farther back than his master, to whom he seems anxious to offer every well-deserved homage.

There is an obscurity in the above quotation, which our readers will not require us to develope, as they have the whole before them. Does this *splendid discovery* consist in the application of the operation by solution, as it is called, to infant subjects? This we are ready to attribute to Mr. Saunders. Is it a discovery that this operation, which Mr. Saunders, we believe, first ventured to perform on a very young subject, was, by his successor, performed on an adult! or that it "*was* exclusively applicable to the congenital and soft or flocculent cataract?" Lastly, does this mean that soft or flocculent cataracts in adults are to be treated in one way if congenital, and in another if commencing after birth?

This paper concludes with some remarks on Sir William's pretensions, to enumerate which, or even the pretensions themselves, would, we conceive, only fatigue our readers.

An Appendix follows, containing several letters, which afford so little information, that they might be all very well omitted. Subjoined to this appendix, is a paper called a "Statement;" in the course of which we find that it is the intention of the governors of the Eye Infirmary to enlarge their plan, by procuring a freehold building for their purpose; and "that, their intention, as a public body, being only that of increasing a public benefit to the full extent of which it admits, an application to government to aid this important design has been for some time contemplated."

Now this at once explains why Sir William, who understands puffing as well as other people, must be laid aside. But, if we are to have an establishment supported by government, should not the officers confine themselves exclusively to that department? What other advantage can such a foundation offer? If the medical officers are to be surgeons of the other royal hospitals, can they not practice and teach all they know at those hospitals, without the necessity of new and consequently expensive establishments, and without increasing the expense of a medical education? No one can be more ready than ourselves to admit that operations on, and even examinations of, so delicate and complex an organ as the eye, are likely to be more perfect when confined to those who are unembarrassed by other occupations, or whose

 manipulations manipulations are never directed to larger and less complicated organs. In a word, if the object of this report was only to show the insufficiency of Sir W. A.'s pretension, that might have been done with equal facility and brevity. If to solicit the aid of government, or private benevolence, a more direct mode would have, in our opinion, proved more successful.

---

*Edinburgh Medical and Surgical Journal*, No. XLI. for January 1815.

*(Continued from p. 228.)*

Art. IX.—*A Reply to a Letter on the Treatment of Fever by Blood-letting, addressed by the four Permanent Physicians of the Committee of the Fever Hospital, Cork-street.* By Thomas Mills, M.D. &c.

As far as we can judge at this distance from the scene of action, the same business that we once witnessed with Dr. Jackson and the Army Medical Board, seems to have been transacted again at Cork. The necessity of blood-letting in fevers is now so well established, that Dr. Mills cannot fear of having partisans every where but at home. There, perhaps, he may share the fate of other prophets. We cannot help expressing concern at the perusal of the paper, on account of the too great sensibility with which it is dictated. We sincerely hope that our few remarks may prove some relief to the author's feelings.

Art. X.—*Letter to the Editors of the Edinburgh Medical and Surgical Journal, from the Medical Officers of Greenwich Infirmary, vindicating Sir William Adams from the charge of keeping secret his Improved Operations on the Eye; with some Observations by the Editor.*

Perhaps the vulgar may say " it's all my eye," Sir William Adams is a knight; he has written, and induced others to write, several things to prove that he is the greatest of all possible oculists. Can he suppose that no one will take offence at this? Have not other surgeons, from a sister kingdom, written on the eye? Have we not also in London an institution, the medical officers of which, though engaged in general practice, conceive that all eye practice should be centered in their establishment? We mean not, however, to undervalue the Editor's reply, but perfectly agree with him, that whatever communication was made by Sir William, was not to the public but to the board, which acknowledges its obligation.

Art. XI.—*Case of Compound Fracture of both Legs, successfully Treated.* By John Peake, Member of the Royal College of Surgeons.

Art.

Art. XII.—*Case in which a Mass, resembling a Placenta without a Fœtus, was discharged from the Womb.* By M. Lemon, Member of the Royal College of Surgeons in London.

This paper is of considerable importance; and, if the appearances are not related with that perspicuity which we could wish, we must impute it to the difficulty of the attempt, rather than to any incapacity in the writer. However, as every hint may be useful towards the future examination of such ill-defined substances, we shall follow the example of the Edinburgh editor, first transcribing the case, and then offering all that occurs to us on the question.

"Mrs. Cunningham, æt. 24, has had three children, besides a miscarriage, which happened about two years ago. The extreme rudeness of a licentious fellow having, on Thursday the 11th of August, required her to make violent exertion, she, on the following day, perceived a slight flow of blood from the vagina, which ceased on Saturday evening. On Sunday night, when in bed, it returned with increase, and continued (gradually diminishing) till Wednesday. All this time she suffered no pain, nor was she prevented from managing her domestic affairs—there was a slight tenderness of the abdomen only. After having had two or three pains, in every respect like those experienced during the time of parturition, she, about half-past five in the evening, was seized with a profuse hæmorrhage. The pains and hæmorrhage continuing, my immediate attendance, for the first time, was requested. It was half-past nine when I saw the patient, who told me she had advanced near four months in her pregnancy; and that the discharge was alarming only during the presence of a pain. Examining *per vaginam,* I found the *os uteri* dilated to the extent of a half-crown: and a bag, protruding through it, distended, as I thought from its elastic feel, with the liquor amnii. About eleven o'clock, a fleshy cake, three inches in diameter, possessing every character of a natural placenta, and having a membranous bag connected with it, was expelled. The shape of this mass was oblong. Slitting up the bag,* which was then flaccid, the contents gave an appearance (not with regard to colour, similar to that which is presented on the exposure of the thoracic and abdominal viscera of a very young fœtus. But the expansion of the placenta rendered the nature of this appearance evident. Its whole surface was covered with tumours. There were about twenty-two distinct, besides many inconsiderable ones, of various size, shape, and colour; some in clusters, (with broad bases) all seemingly connected by veins. The largest tumour was equal in magnitude to a small walnut; but this was easily, though not completely, divisible into two. Many were of the size of grapes; some were flat-sided;

"* There was no fluid found when the bag was cut into."

others others conical, oblong, irregular, and convoluted. Most were livid; some fleshy-brown, and two or three light yellow. There was one, larger than the rest, semi-transparent, membranous, and globular, with its base surrounding the largest of the latter.* This appeared to be formed by the raising of the covering of the fœtal surface of the placenta, which, after affording a tunic to all the tumours, was continued from the circumference of the placenta to form the bag. This membranous continuation had another imperfect fatty layer proceeding from the uterine surface of the placenta. The livid tumours generally had condensed fat at the extremity. All had the same feel, except the yellow, which consisted of a compact fatty substance, resembling that found on the other side of the placenta. The livid and brownish had not the firmness of fleshy tumours, nor the elasticity of those containing fluid. They contained coagulated blood; and, pressing it out, a substance of a placental nature was left betwixt the fingers. Such is my idea of these tumours, that, were my opinion of the formation of them demanded, I should say it was probable that the blood, being extravasated from the vessels of the placenta, or having accumulated in its cells, that tuberculated surface was produced by an irregular and great distention, or, perhaps, rupture of them. Notwithstanding, I presumed it was evident that some of the veins, or sinuses of the placenta, had degenerated into these tumours, as their regular progression could apparently be traced.

"The woman, during the growth of this mass within the uterus, had every symptom of pregnancy. The breasts, when she is not pregnant, are scarcely observable; now, they are rather prominent, and distinctly marked. About the third month of gestation, a soreness was always produced by pressure; this she felt at this time, under the same circumstances. She had the same feelings, the same nausea, and the same capricious appetite, she always had when with child. There was a prominent firmness of the belly, and a total disappearance of the menses. At the time she was taken ill, she expected to quicken; but she yet had not felt any internal motion.

"Here are the most satisfactory symptoms that accompany a natural and common conception, yet not even the rudiments of a child have, by the strictest examination, been discovered. The bag in which the child, if it had existed, would have been found, was, as far as I could judge from a particular inspection, perfectly entire. It, therefore, cannot be consistently conjectured that the child might have escaped at the time of the flooding.

"That the womb had not contained a fœtus, even just before the threatening of the supposed abortion, is proved beyond doubt, by there not being the remains of a funis, or by the mark of its

"* I cannot say whether this vesicle contained air or water, as it was relaxed before I could attend to it."

attachment not existing. Nature, very likely, intended to have created a child, but, whether the embryo had been formed, and afterwards dissolved, I do not know; nor, indeed, have I at this moment leisure to prosecute the inquiry with sufficient steadiness: I shall, therefore, leave it to the ingenuity of others.

"The 9th plate in Denman's 4to. edition, published in 1805, gives a pretty correct representation of the body I have endeavoured to describe.

"I have searched Morgagni, and many other celebrated authors, with some degree of diligence; yet not one case have I been able to find exactly correspondent to this. Every case that throws light upon medical jurisprudence, must, by every well-wisher of mankind, be considered highly important, not only in a judicial, but in a moral and political point of view. Our present knowledge of it is very limited; our ideas unsettled, and indefinable. The works written professedly upon this subject, are either too confined in their plan, or too speculative in their execution. Cautious deductions, drawn from absolute facts, or facts themselves, should constitute a practical system of medical jurisprudence.

"Those characters by which we are taught to distinguish a uterus lately impregnated, are, a ruggedness of a certain extent, according to the time of gestation of its internal surface,—the size of its cavity,—the thickness of its walls,—and the largeness of its mouth. But, upon the first of these, the greatest stress is laid. I seriously ask you, then, would not the womb of my patient have exhibited the same characters? Now, sir, had this woman died from hæmorrhage, and had her death been made a subject of legal investigation; and had I been desired to examine the body, without having any previous knowledge of the person, what evidence must I have given? Had I doomed an innocent fellow-creature to eternal infamy by rashly swearing that a child had been recently expelled from the womb,—from the placental mark, from the capacity of the cavity of the uterus, and from the dilatation of its mouth,—I should have been supported by gentlemen who are justly regarded as some of the brightest ornaments in the medical world. The mark of a placenta has, by very eminent practitioners, been insisted on as 'an indubitable proof of the existence of a child.' I most sincerely wish, for the good of society, it could be proved to be so. I am not ashamed to confess that I am totally unacquainted with any circumstance, which, with certainty, points out the difference in this womb from a womb having contained a fœtus. But perhaps the kindness of some of your correspondents will favour me with information on this subject, which will much oblige, &c."

The first inquiry we would make is the nature of the injury, whether likely to be sufficient to induce miscarriage? Next, as the patient had miscarried before, whether about the same period of supposed pregnancy, and what was the appearance of the abortion? We may next remark, that, whatever may have been the nature of the substance in the present instance, the short period between the injury received and the discharge, prevents any suspicion that the first could have influenced the nature of the second. The various forms described as composing the contents of the sac, are such as frequently occur where hydatids are discovered; and scarcely a description of steatomatous tumours is unattended with other cysts filled with different substances. Dr. Adams, in his "Treatise on the Cancerous Breast," has shown the almost universal connection of these substances with each other; and does not scruple to assign to them the same origin and the same laws. We say nothing about the too common expression of blood-vessels of any kind "*degenerating* into these tumours." We can only describe them as we see them; and, if we must use an expression consistent with our opinion, it would be that the cysts were found in different stages of growth or maturity, attached to each other, or to the blood-vessels.

[The quantity of original matter which has pressed upon us lately, has prevented the insertion of any review of SPURZHEIM'S Craneology for the last month.]

